# The Relative Value of Fillings

**Published:** 1898-06

**Authors:** C. P. Pruyn


ARTICLE IV.

### THE RELATIVE VALUE OF FILLINGS.

DR. C. F. PRUYN.

The relative value of filling material depends largely on the individual using them, and the conditions under which they are used. Gold is the best, but there are other filling materials that are very valuable to us, and they should not be decried because they are sometimes badly or carelessly used. I allude particularly to amalgam. Amalgam has a valuable place in dentistry, and its particular value to me appears to be in a combinaion of the two metals, gold and amalgam, in the same tooth. Which is the better of the two, is a hard matter to say. We might use an illustration of this kind: Which is of the greatest importance in the propagation of the human species, man or woman? Neither is complete without the other. Which is the better of the two, gold or amalgam? When the two are used in combination they fill a field of usefulness that neither one of them fills alone. This is my honest belief. This is a belief that comes to me from an experience of nearly twenty-five years in the practice of dentistry, and for twenty years in this particular line of practice. Take, if you please, extensive decay on the distal surfaces of bicuspids and molars; fill such cavities with amalgam; at the succeeding sitting fill the crown portion of the cavity with gold, so that the two metals may be in complete opposition, and thus no electrical energy is generated to any appreciable extent. After a short time the baser metal will become oxidized, in most cases, beautifully ebonized, the pure metal will be kept bright and clean, the edges of the amalgam filling will have a much better appearance than if the amalgam was used alone because of the oxidation of the baser metal. So that in my practice I warmly advocate the use of the combination of gold and amalgam in the same tooth. Be sure to have the two metals in apposition. By following this line of practice the amalgam is placed at the cervical borders, where it exhibits its greatest usefulness, while gold will do its best work on the occlusal surfaces, as an illustration we have here, two valuable members of society, each one having its particular position in society where it can be of the most service.

The greatest failure of gold occurs on the cervical borders, and amalgam on the surface. Now by putting each one where it would be at its best, we can do better work than with either one of the metals alone. I frequently see such fillings that have been in the mouth twenty years doing good service to-day, and I feel sure that I am saving with this method of practice more teeth than I could do with the use of either one of these metals separately. It took me a little time to become a convert to this theory, but after consulting some of the old practitioners who had followed this plan for some years, I saw in it a field of usefulness. I have made a great many converts among my professional acquaintances who have carefully observed the results of this line of my practice.

Twenty years ago or more we were startled by a new departure, a new theory, as some of you remember. There was a triumvirate in this country, consisting of Drs. Flagg, Palmer and Chase, who said that gold was the worst material to use, and that as teeth needed saving amalgam was the best thing to use. It startled us. It came to us "like thunder out of a clear sky," and we began to investigate a little to see whether there was any truth in this wild vagary. As we examined into it we found there was a grain of truth, and that grain of truth was worth investigating. I am thoroughly convinced that I could hardly use either gold or amalgam and say this is the thing par excellence; but I place them hand in hand for the preservation of teeth. I can hardly say definitely and clearly that one of these two is better than the other, because each one has its own particular field of usefulness, and the relative value of each one depends on its environments and to a considerable extent on the thoroughness with which it is manipulated.

The other materials, gutta percha and cement, of course have a field of usefulness, but to my mind a much more limited one for permanence than either of the two mentioned.—*Dental Review.*

